# CT-based determination of excessive visceral adipose tissue is associated with an impaired survival in critically ill patients

**DOI:** 10.1371/journal.pone.0250321

**Published:** 2021-04-16

**Authors:** Theresa H. Wirtz, Sven H. Loosen, Maximilian Schulze-Hagen, Ralf Weiskirchen, Lukas Buendgens, Samira Abu Jhaisha, Jonathan F. Brozat, Tobias Puengel, Mihael Vucur, Pia Paffenholz, Christiane Kuhl, Frank Tacke, Christian Trautwein, Tom Luedde, Christoph Roderburg, Alexander Koch

**Affiliations:** 1 Department of Medicine III, University Hospital RWTH Aachen, Aachen, Germany; 2 Clinic for Gastroenterology, Hepatology and Infectious Diseases, University Hospital Düsseldorf, Medical Faculty of Heinrich Heine University Düsseldorf, Düsseldorf, Germany; 3 Department of Diagnostic and Interventional Radiology, University Hospital RWTH Aachen, Aachen, Germany; 4 Institute of Molecular Pathobiochemistry, Experimental Gene Therapy and Clinical Chemistry, University Hospital RWTH Aachen, Aachen, Germany; 5 Department of Hepatology and Gastroenterology, Charité University Medicine Berlin, Berlin, Germany; 6 Department of Urology, University Hospital Cologne, Cologne, Germany; BronxCare Health System, Affiliated with Icahn School of Medicine at Mount Sinai, NY, USA, UNITED STATES

## Abstract

**Objective:**

Obesity is a negative prognostic factor for various clinical conditions. In this observational cohort study, we evaluated a CT-based assessment of the adipose tissue distribution as a potential non-invasive prognostic parameter in critical illness.

**Methods:**

Routine CT-scans upon admission to the intensive care unit (ICU) were used to analyze the visceral and subcutaneous adipose tissue areas at the 3^rd^ lumbar vertebra in 155 patients. Results were correlated with various prognostic markers and both short-term- and overall survival. Multiple statistical tools were used for data analysis.

**Results:**

We observed a significantly larger visceral adipose tissue area in septic patients compared to non-sepsis patients. Interestingly, patients requiring mechanical ventilation had a significantly higher amount of visceral adipose tissue correlating with the duration of mechanical ventilation. Moreover, both visceral and subcutaneous adipose tissue area significantly correlated with several laboratory markers. While neither the visceral nor the subcutaneous adipose tissue area was predictive for short-term ICU survival, patients with a visceral adipose tissue area above the optimal cut-off (241.4 cm^2^) had a significantly impaired overall survival compared to patients with a lower visceral adipose tissue area.

**Conclusions:**

Our study supports a prognostic role of the individual adipose tissue distribution in critically ill patients. However, additional investigations need to confirm our suggestion that routine CT-based assessment of adipose tissue distribution can be used to yield further information on the patients’ clinical course. Moreover, future studies should address functional and metabolic analysis of different adipose tissue compartments in critical illness.

## Introduction

Obesity is defined as a condition of excessive fat accumulation. It is associated with an increased risk for severe diseases including malignancies and cardiovascular-related health problems [1, 2]. In critical illness, severe obesity augments the risk of complications in several organ systems. However, a moderate overweight and mild obesity are associated with a lower mortality in patients treated on medical intensive care units (ICU) [3]. This phenomenon is referred to as the “obesity paradox” and has also been observed in other chronic diseases including coronary artery disease [4] and end-stage kidney disease [5].

In general, obesity is quantified based on the patient’s body mass index (BMI). However, critically ill patients are exposed to several influences that impact the body composition, such as an excessive fluid overload or sarcopenia. Moreover, patients with a similar BMI may differ with respect to the individual adipose tissue distribution [6]. A patient´s individual adipose tissue composition (e.g. visceral and subcutaneous adipose tissue) therefore complements the diagnosis of obesity and both tissue compartments have previously been shown to correlate with obesity determined by BMI [7, 8]. The adipose tissue distribution moreover is of prognostic relevance, since the different compartments of adipose tissue are characterized by distinct metabolic processes with either positive or negative effects on the clinical course of critically ill patients. For example, the visceral adipose tissue as well as ectopic fat–e.g. abnormal fat deposition in the liver, the muscles or the heart–was referred to as “sick fat” [9, 10]. In contrast, subcutaneous fat tissue was reported to cause rather advantageous effects–a finding referred to as “metabolically healthy obesity” [11, 12].

Since the BMI is a poor marker to indicate fat tissue mass and -distribution, further tools are required to more specifically assess the individual ICU patient’s body composition. In this context, routine radiological scans could be helpful and furthermore are easily accessible as they represent a common diagnostic feature in critically ill patients. In this study, we used routine CT-scans performed at ICU admission to analyse the visceral and subcutaneous adipose tissue area. We hypothesized that this imaging-guided analysis could help to explain obesity-related influences on the clinical course of critical illness and to improve prediction of prognosis.

## Patients and methods

### Study design and patient characteristics

155 patients admitted to the ICU of the Medical Department III at the RWTH Aachen University Hospital between 2006 and 2015 were enrolled in our observational cohort study (see Table 1 for detailed patients´ characteristics). Only patients with an age of ≥18 years, an expected ICU stay of >72h and who received an CT-scan at ICU admission were included in our study. Written informed consent was obtained from all patients or authorized relatives / legal guardians in the case of unconsciousness. The study and specifically the consenting procedure were approved by the local ethics committee (EK 150/06) of the RWTH Aachen University Hospital, Germany, and conducted according to the ethical standards laid down in the Declaration of Helsinki. Patients´ follow-up and analysis of long-term mortality was performed up to two years after study inclusion.

**Table 1:** Baseline patient characteristics at the time point of admission and mean visceral and subcutaneous adipose tissue areas.

Parameter	Patients
ICU patients	155
Gender	
Female (%)	39.4
Male (%)	60.6
Age, median in years (range)	60 (21–88)
BMI [kg/m^2^], median (range)	25.2 (12.9–69.9)
Diabetes mellitus type 2 (%)	23.9
Arterial hypertension (%)	44.5
Coronary artery disease (%)	21.9
Malignancy (%)	28.4
Main diagnosis/reason for admission (%)	
Sepsis	50.4
Infectious focus of sepsis (%)	
Pulmonary	50.0
Abdominal	15.4
Urinary tract	10.3
other	24.3
Cardiopulmonary disease	13.5
Acute liver failure	4.5
Liver cirrhosis	7.1
Acute pancreatitis	5.2
Gastrointestinal bleeding	7.7
Other	11.6
APACHE-II score at day 1	23 (3–47)
SOFA score at day 1	11 (0–19)
SAPS-II score at day 1	47 (8–86)
Mechanical ventilation demand (%) at day 1	67.7
Stay on ICU, median in days (range)	11 (1–399)
Death on ICU (%)	28.4
30d mortality (%)	26.5
90d mortality (%)	43.9
180d mortality (%)	51.0
365d mortality (%)	60.0
long-term mortality (%)	74.8
Visceral adipose tissue area, cm^2^ (range)	134.4 (3.16–581.5)
Subcutaneous adipose tissue area, cm^2^ (range)	171.0 (6.56–741.6)

For quantitative variables, median and range (in parenthesis) are given. *Abbreviations are*: BMI, body mass index; APACHE, Acute Physiology and Chronic Health Evaluation; SOFA, sequential organ failure assessment; SAPS-II, simplified acute physiology score; ICU, intensive care unit.

### Assessment of adipose tissue composition

**Fig 1:**
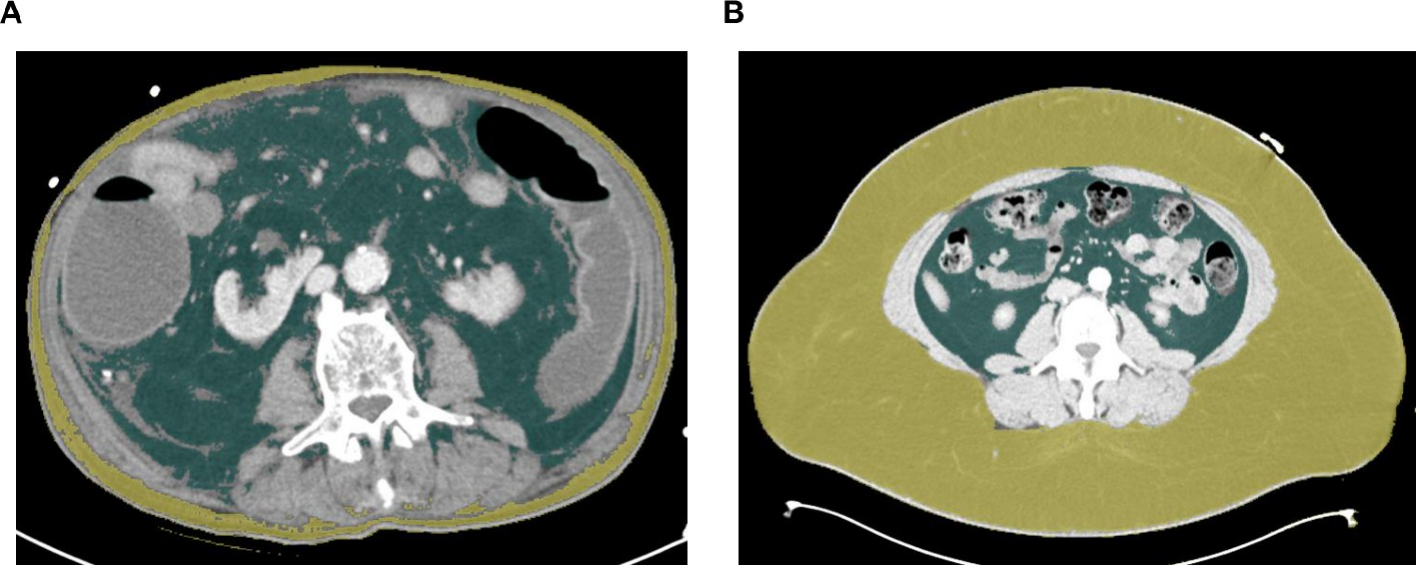
Assessment of visceral and subcutaneous adipose tissue distribution. The visceral and subcutaneous adipose tissue areas were segmented at the center plane of the 3^rd^ lumbar vertebra on axial CT-scans using a semi-automatically segmentation tool (3D slicer) [13]. (A) An exemplary scan of a patient with excessive visceral (green area) and low subcutaneous (yellow area) adipose tissue is depicted. (B) An exemplary scan of a patient with low visceral (green area) and excessive subcutaneous (yellow area) adipose tissue is depicted.

CT-scans in venous phase with a slice thickness of 5 mm that were performed during clinical routine upon ICU admission were used for this analysis. We manually assessed the adipose tissue distribution using the semi-automatical segmentation tool “3D slicer”, an open source software platform for medical image informatics [13]. Both the visceral adipose tissue (VAT) and the subcutaneous adipose tissue (SAT) were segmented at the center plane of the 3^rd^ lumbar vertebra on axial CT-scans (Fig 1). This CT plane was chosen to gain the highest comparability with existing studies [14, 15]. Participants with missing data were omitted from the analysis.

### Measurements of standard laboratory parameters and adiponectin serum concentrations

Standard laboratory markers were available for the total amount of 155 patients and were analyzed in the central laboratory at University Hospital RWTH Aachen using a Sysmex XN9000 (Sysmex GmbH, Norderstedt, Germany) and a Cobas 8000 c701 (Hoffmann-La Roche AG, Basel, Switzerland) platform according to the manufacturer’s instructions. For a small subset of patients (n = 17) serum samples upon ICU admission were available for serum concentration determination of markers beyond the clinical routine including adiponectin. Peripheral or central venous blood was obtained at ICU admission prior to any therapeutic intervention. Blood samples were centrifuged (2,000 x g for 10 minutes) and serum samples were stored at −80°C until use. Serum adiponectin levels were analyzed in a blinded fashion by an enzyme-linked immunosorbent assay (ELISA) according to the manufacturer’s instructions (No. EZHADP-61K, MERCK Millipore, Schwalbach, Germany).

### Statistical analysis

Statistical analyses were performed as recently described [16]. Before initiation of analysis, a power calculation using Power and Sample Size Program, Version 3.0, January 2009, was conducted revealing an adequate power of >0.80. Shapiro-Wilk test was used to test for normal distribution. Mann-Whitney-U-Test and Kruskal-Wallis-Test were used to compare non-parametric data between two or more groups. Kaplan-Meier curves display the impact on overall survival as primary study outcome. The Log-rank test was performed to test for significance between groups. The optimal cut-off value for the identification of patients with an impaired overall survival was established using a recently published biometric software [17]. The prognostic value of variables was further tested by univariate analysis in the Cox regression model. The hazard ratio (HR) and the 95% confidence interval are displayed. All statistical analyses were performed using SPSS 23 (SPSS, Chicago, IL, USA) [2]. A *p*-value of < 0.05 was considered statistically significant (* *p* < 0.05; ** *p* < 0.01; *** *p* < 0.001).

## Results

### Description of study cohort

A total of 155 critically ill patients admitted to the ICU and who received a CT-scan upon ICU-admission were included into analysis. The median visceral adipose tissue (VAT) area was 134.3 cm^2^ with a range from 3.16 to 581.5 cm^2^, whereas the median subcutaneous adipose tissue (SAT) area was 171.0 cm^2^ with a range from 6.56 to 741.6 cm^2^. Both adipose tissue areas were provided for all included patients. Further detailed patient characteristics are shown in Table 1.

**Fig 2:**
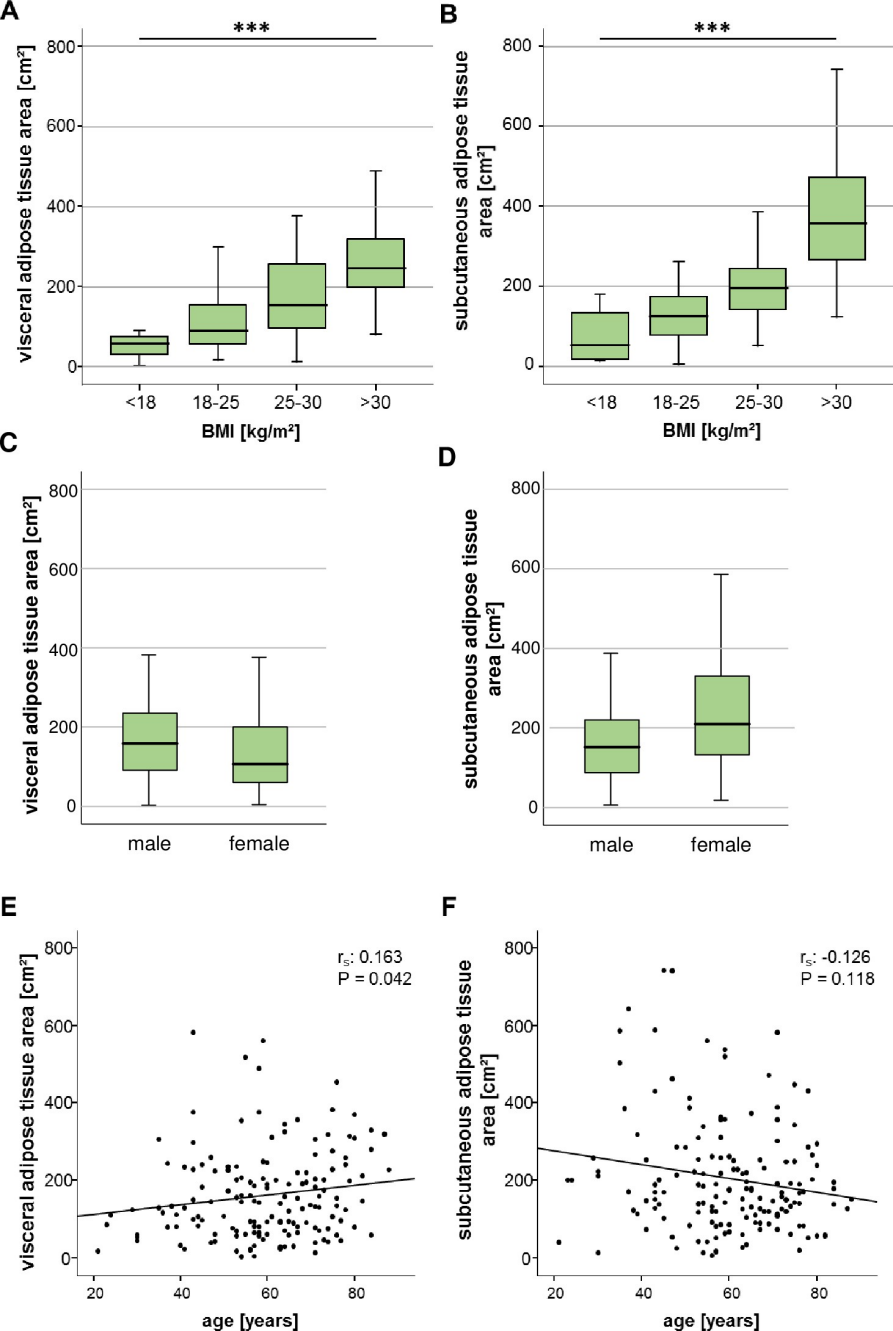
The ICU patients’ visceral and subcutaneous adipose tissue areas correlate with BMI and differ between the genders. (A, B) Both VAT and SAT areas are closely associated with the clinical categories of the patients´ body mass index (BMI). (C) Female ICU patients are characterized by a significantly smaller VAT area compared to male patients, whereas the SAT area is increased in female compared to male patients (D). (E) The patients´ age positively correlates with the VAT area but not the area of the SAT (F). Data are expressed as correlation analyses including Spearman´s rho coefficient (A, B, E, F) or means ± SEM (C, D); *p<0.05, p***<0.001.

We first analysed whether the VAT and SAT area correlate with demographic and clinical baseline characteristics of critically ill patients. Both the VAT as well as the SAT area were closely associated with the clinical categories of the body mass index (BMI), that are defined as underweight (<18 kg/m^2^), normal weight (18–25 kg/m^2^), overweight (25–30 kg/m^2^) and obesity (>30 kg/m^2^) (Fig 2A and 2B). The VAT area was decreased in female compared to male patients, whereas female patients had a significantly higher SAT area compared to male patients (Fig 2C and 2D). At higher age, patients displayed increasing areas of VAT, but not SAT (Fig 2E and 2F).

### VAT and SAT areas are associated with cardiovascular risk factors and correlate with markers of organ dysfunction in critically ill patients

**Fig 3:**
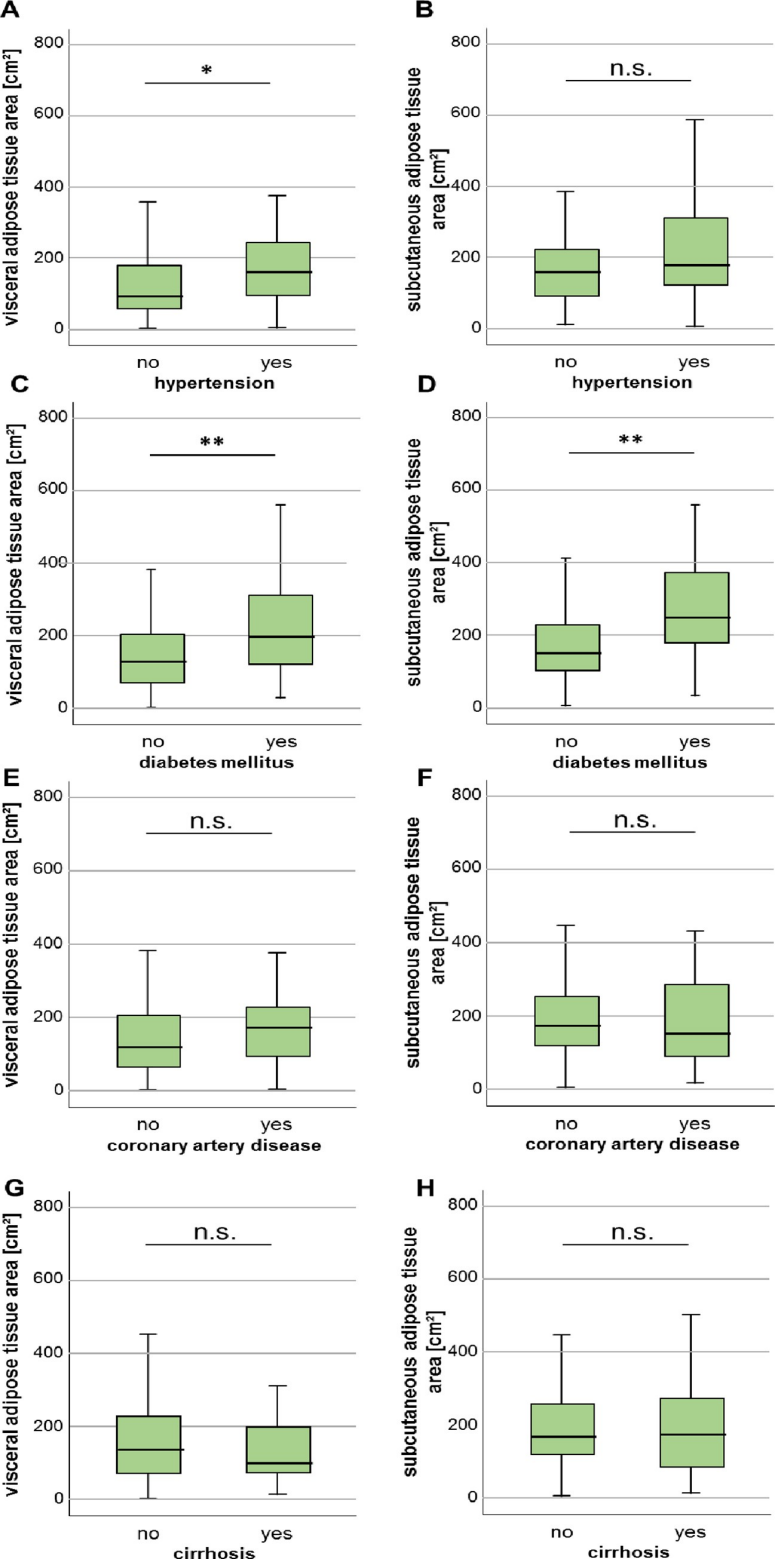
Characteristics of visceral and subcutaneous adipose tissue area in ICU patients with various comorbidities. (A) The VAT area is increased in ICU patients with arterial hypertension. (B) In contrast to the SAT area does not differ between patients with and without arterial hypertension. (C, D) Both the VAT as well as SAT area is significantly increased in patients with diabetes mellitus type 2. Patients with previous diagnosis of coronary artery disease (E, F) or liver cirrhosis (G, H) do not show differences in VAT or SAT areas. Data are expressed as or means ± SEM; *p<0.05, **p<0.01.

Next, we investigated whether existing disease conditions are associated with differences in areas of VAT and SAT. Patients with arterial hypertension had an enlarged area of VAT, whereas the SAT area did not differ between patients without or without arterial hypertension (Fig 3A and 3B). Diabetes mellitus type 2 was associated with both an increased VAT and SAT area compared to non-diabetic patients (Fig 3C and 3D). However, previous diagnosis of coronary artery disease did not influence the measured areas of both VAT as well as SAT (Fig 3E and 3F). Other medical conditions, i.e. liver cirrhosis (Fig 3G and 3H), chronic obstructive pulmonary disease (COPD), end-stage kidney disease with dialysis as well as frequent alcohol intake did not correlate with differences in adipose tissue areas (S1 Fig in S1 File).

Since bacterial infection and organ dysfunction are common in critically ill patients, we evaluated whether adipose tissue areas correlate with markers of systemic inflammation or organ dysfunction. First, neither the VAT nor the SAT area correlated with systemic inflammation indicated by the C-reactive protein (CRP), interleukin 6 (IL-6) or bacterial infections indicated by procalcitonin (PCT) (Table 2). However, the SAT, but not the VAT area was associated with increasing leukocyte counts. Regarding organ dysfunction, none of both adipose tissue areas correlated with kidney function parameters including creatinine or urea. The SAT area strongly correlated with bilirubin, aspartate-aminotransferase (AST), alanine-aminotransferase (ALT) and also gamma-glutamyltransferase (yGT), whereas the VAT area did not correlate with any of these liver function parameters. Regarding glucose metabolism, larger VAT areas went along with higher glucose serum levels and also higher insulin concentrations. Both adipose tissue areas positively correlated with the glycosylated hemoglobin (HbA1c). In a subset of 17 patients, the adipocyte-derived hormone adiponectin was measured and negatively correlated with the VAT area. Lastly, the investigated adipose tissue areas did not correlate with clinical scoring systems for critical illness (APACHE II, acute physiology and chronic health evaluation score; SOFA, sepsis-related organ failure assessment *score;* SAPS II, simplified acute physiology score).

**Table 2:** Correlations of VAT and SAT area with markers of inflammation, markers of organ dysfunction, metabolic laboratory markers and clinical scores at day 1 of ICU admission.

	VAT area		SAT area	
	r	p	r	p
**Markers of inflammation**				
Leukocytes	0.088	0.283	0.176	0.031*
CRP	0.117	0.151	0.068	0.405
Procalcitonin	0.091	0.316	0.082	0.371
IL-6	0.120	0.176	0.006	0.944
**Markers of organ dysfunction**				
Creatinine	0.039	0.630	-0.024	0.769
GFR	-0.029	0.735	0.022	0.769
Urea	-0.011	0.892	-0.088	0.284
Sodium	0.140	0.084	-0.023	0.776
Kalium	0.060	0.459	-0.012	0.879
Bilirubin total	0.081	0.327	0.188	0.022*
AST	0.041	0.629	0.288	<0.001***
ALT	0.010	0.900	0.214	0.008**
yGT	0.140	0.085	0.189	0.020*
NT-proBNP	-0.052	0.581	-0.096	0.308
LDH	-0.026	0.747	0.254	0.002**
Lactate	0.096	0.246	0.164	0.046*
**Glucose and fat metabolism**				
Glucose	0.177	0.040*	0.004	0.965
Insulin	0.592	0.008*	0.454	0.051
HbA1c	0.548	0.006*	0.405	0.049*
Cholesterin	0.157	0.083	0.098	0.283
Adiponectin	-0.615	0.009**	-0.400	0.112
Leptin	0.194	0.456	0.350	0.168
Somatotropin	-0.231	0.389	-0.511	0.043*
Cortisol	-0.637	0.004**	-0.558	0.016*
**Clinical scores**				
APACHE II	0.142	0.238	0.068	0.576
SOFA	0.146	0.287	0.132	0.340
SAPS II	0.227	0.176	0.171	0.319

Spearman rank correlation test was used to test significance; the Spearman´s rho correlation coefficient is depicted as “r” with *p<0.05

**p<0.005

***p<0.001. *Abbreviations*: VAT, visceral adipose tissue; SAT, subcutaneous adipose tissue; BMI, body mass index; CRP, C-reactive protein; GFR, glomerular filtration rate; AST, aspartate aminotransferase; ALT, alanine aminotransferase; yGT, gamma glutamyltransferase; LDH, lactate dehydrogenase; BNP, brain natriuretic peptide; HbA1c, glycosylated hemoglobin; APACHE II, acute physiology and chronic health evaluation score; SOFA, sepsis-related organ failure assessment *score;* SAPS II, simplified acute physiology score.

### Excessive VAT is associated with a more severe clinical course of critically ill patients

**Fig 4:**
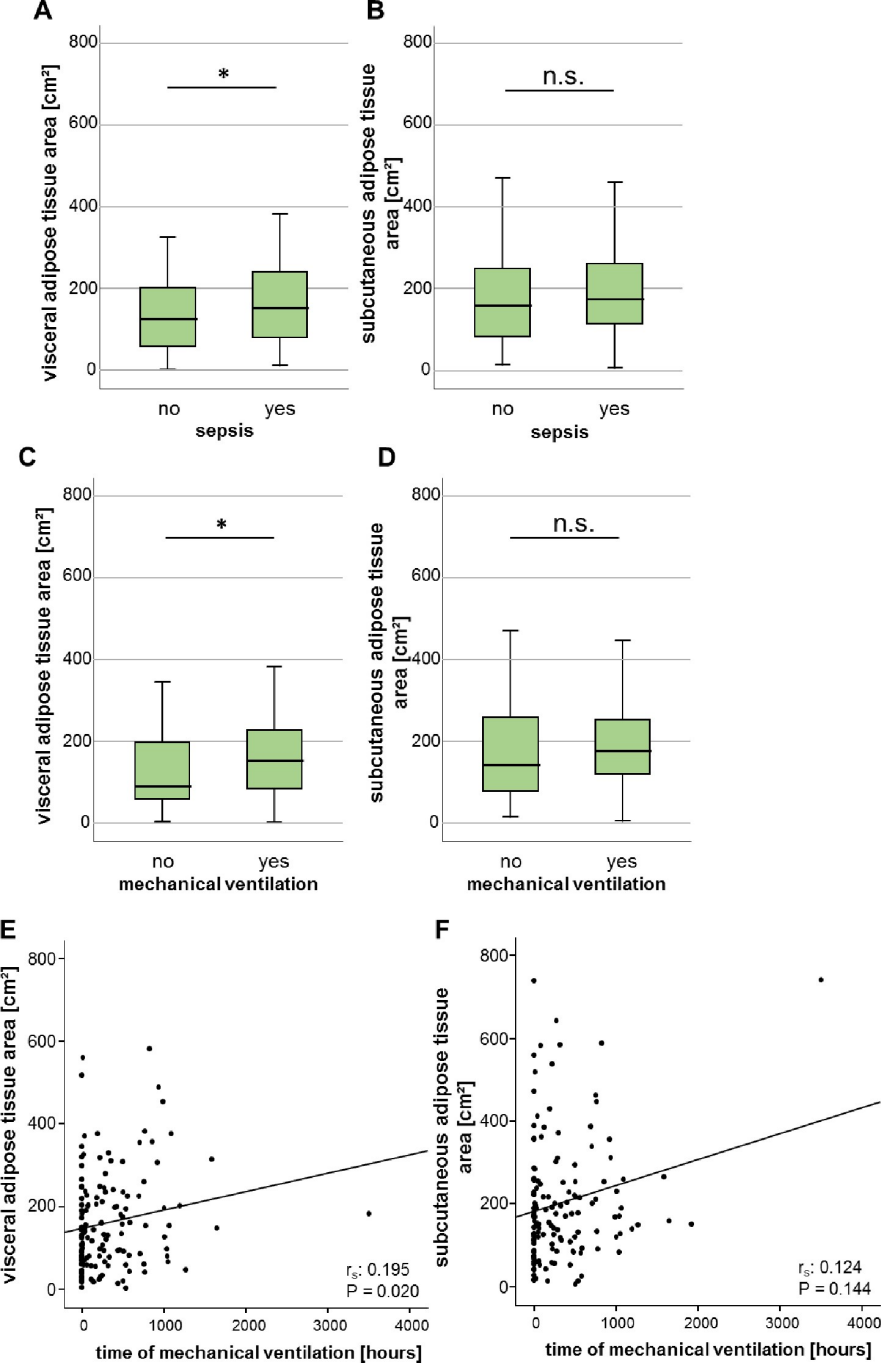
Augmented areas of the VAT are associated with increased duration of mechanical ventilation and prevalence of sepsis in critically ill patients. (A, B) Sepsis patients show an increased area of VAT but not SAT area compared to non-sepsis patients. (C, D) ICU patients requiring mechanical ventilation are characterized by a significantly increased area of the VAT but no difference of the SAT area. (E, F) There is a significant positive correlation between the VAT, but not the SAT and the duration of mechanical ventilation. Data are expressed as means ± SEM (A, B, E, F) or correlation analyses including Spearman´s rho coefficient (C, D); *p<0.05.

Since obese patients are characterized by a disadvantageous clinical course in several acute and chronic diseases, we investigated whether the adipose tissue area might reflect the clinical course of ICU patients. Interestingly, patients who were admitted to the ICU due to sepsis were characterized by an enlarged VAT area, whereas the SAT area did not differ compared to non-sepsis patients (Fig 4A and 4B). Moreover, patients with respiratory failure requiring mechanical ventilation showed an increased VAT area, whereas the SAT area was not different compared to patients without mechanical ventilation (Fig 4C and 4D). Furthermore, the measured VAT area positively correlated with the time of mechanical ventilation, but the SAT area did not (Fig 4E and 4F).

### The VAT area is a prognostic factor for overall survival

**Fig 5:**
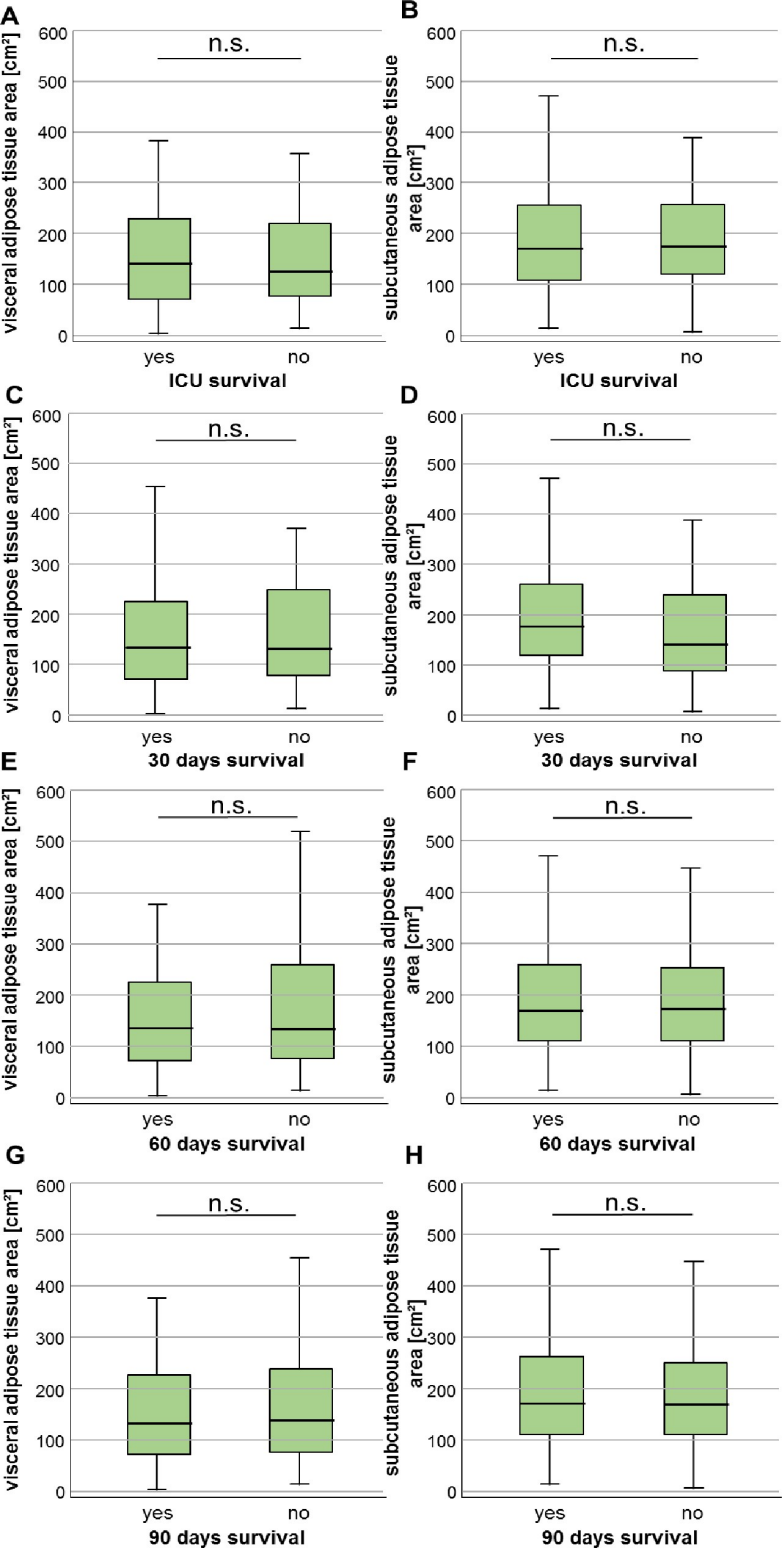
Neither the VAT nor SAT area predicts short-term survival in critically ill patients. (A, B) Patients who survive the ICU stay have similar VAT as well as SAT areas compared to ICU non-survivors. Both tissue areas are unaltered in patients who did or did not survive beyond 30 (C+D), 60 (E+F) or 90 days (G+H) after ICU admission. Data are expressed as or means ± SEM.

We next hypothesized that due to our finding of increased VAT area in sepsis patients and its correlation with duration of mechanical ventilation, the VAT area could correlate with the individual patient’s short- and/or long-term outcome. We therefore compared both adipose tissue areas in patients who survived the ICU stay and were discharged to a standard care ward and patients who deceased on the ICU. Here, we did not observe significant differences in the adipose tissue areas in patients who did or did not survive the ICU stay (Fig 5A and 5B). The evaluation of further short-term mortality time-points (e.g. 30 days, 60 days, 90 days) also did not reveal differences in VAT or SAT areas in patients who did or did not survive the respective time-point (Fig 5C–5H).

**Fig 6:**
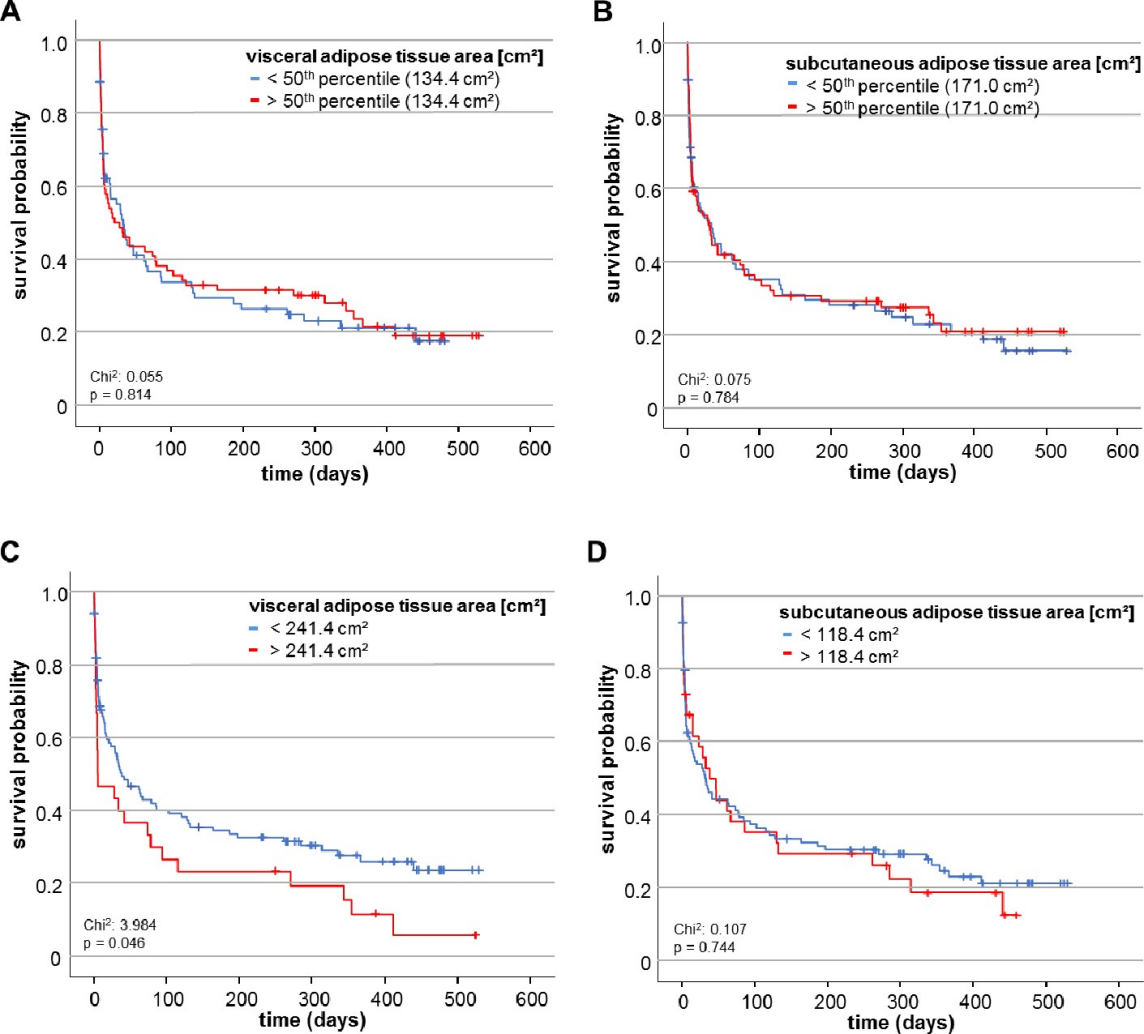
The VAT area predicts overall survival in critically ill patients. (A, B) When using the median of both adipose tissue areas as cut-off values, neither the VAT nor the SAT area cut-off value predicts overall survival as Kaplan Meier curve analysis reveals. (C) Using the optimal cut-off value, patients with a VAT area below 241.4 cm^2^ show an improved overall survival. (D) In contrast to the VAT area, the optimal cut-off value of the SAT area does not differ between overall survivors and non-survivors.

Based on these results we asked whether an increased adipose tissue area might correlate with the overall survival (OS). If so, the prognostic impact of VAT and SAT area determination could exceed the prognostic relevance of the patients´ BMI where we did not reveal differences in overall survival between patients with a BMI above or below the cut-off of 30kg/m^2^ as Kaplan-Meier curve analysis revealed (S2 Fig in S1 File). Therefore, we dichotomised both variables and compared the OS in patients with high or low VAT and SAT areas. Using the median cut-off value of the VAT (134.4 cm^2^) or SAT area (171.0 cm^2^), Kaplan-Meier curve analysis revealed no difference between patients with adipose tissue areas below or above these cut-offs (Fig 6A and 6B). We thereafter acquired an ideal prognostic cut-off value (see Materials & Methods for details). Here, critically ill patients with a VAT area above 241.4cm^2^ had a significantly impaired OS compared to patients with a VAT area below this cut-off value (Fig 6C). The median OS for patients with a VAT area above 241.4cm^2^ was only 6 days compared to 39 days for patients who had a VAT area below the optimal cut-off value.

To moreover stress the prognostic value of VAT area we subsequently performed univariate Cox-regression analysis. Here, a VAT area above the ideal cut-off value of 241.4cm^2^ was identified to represent a significant predictive factor for overall mortality (HR: 1.553, 95% CI: 1.003–2.402, *p* = 0.048). However, the ideal cut-off of SAT area of 118.4cm^2^ was not able to predict overall survival (Fig 6D).

## Discussion

Considering the limitations of determining the fat tissue distribution and body composition in critically ill patients, we here used routine abdominal CT-scan for estimating quantity of adipose tissue in patients treated for critical illness on a medical ICU. Importantly, we demonstrate that patients with a VAT area above an optimal cut-off (241.4 cm^2^) displayed an impaired overall survival compared to patients with a VAT area below this cut-off. Moreover, presence of sepsis was associated with an enlarged VAT area compared to non-sepsis patients. Finally, patients requiring mechanical ventilation had a significantly larger area of the VAT that furthermore correlated with duration of mechanical ventilation.

Obesity is defined as an abnormal or excessive fat accumulation that presents a risk to health, according to the World Health Organization (WHO). Overweight and obesity are major risk factors for a number of chronic diseases, including diabetes, cardiovascular diseases and cancer [18, 19]. Paralleling the epidemic rise of overweight and obesity in the general population, patients with obesity comprise a significant proportion of the critically ill population, estimated to be around 34% for overweight (BMI 25 to 29.9 kg/m^2^) and 15–20% for obesity (BMI >30 kg/m^2^) [3, 19]. In line, in our cohort, the median BMI was 25.2 kg/m^2^; 52% of critically ill patients fulfilled the criteria for overweight and 23.3% fulfilled the criteria for obesity. However, a BMI-guided classification of patients as overweight or obese was considered to be inaccurate during acute illness, as, for example, the presence of tissue edema from fluid resuscitation might create misclassification of normal weight individuals into the overweight category. Therefore, we manually assessed the adipose tissue composition using the semi-automatically segmentation tool “3D slicer” [13]. Both the VAT and the SAT were segmented at the center plane of the 3^rd^ lumbar vertebra on axial CT-scans. The validity of the applied method is highlighted by the following considerations: In our analysis, female patients showed a reduced VAT but an increased SAT area compared to males–a finding that is in line with previous studies [20]. Another finding striking the validity of the imaging-guided adipose tissue determination is the negative correlation of the adipose tissue area and adiponectin serum levels as it was expected [21]. Both the VAT as well as the SAT area showed close association with the patients´ BMI category–however, we assumed that our analysis would reveal further insights in the prognostic relevance of two distinct adipose tissue compartments that is beyond the prognostic significance of the BMI alone.

For patients with obesity, the already complex intensive care medical treatment is sometimes dramatically complicated by numerous factors: airway management and oxygenation might be challenging, underlying metabolic syndrome and diabetes require special considerations in nutritional support, pharmacokinetics are altered and the risks of acute kidney injury or serious cardiovascular complications are higher, just to name a few [22]. In our cohort, patients requiring mechanical ventilation, patients fulfilling sepsis criteria and patients at a higher age were characterized by pronounced areas of VAT–however, correlation analyses were characterized by an increased dispersion of VAT and a reliable statement of association of VAT area with the investigated parameters cannot be made only due to our data but need further investigation. Nevertheless, we based on these observation hypothesized that the VAT area would inadvertently influence outcome within our cohort of critically ill patients. To investigate the impact of VAT area on overall survival we dichotomized the VAT area variable by using a freely available tool called “*Cutoff Finder*” developed to facilitate cut-off optimization for biomarkers [17]. This tool aims at avoiding both overestimation of the significance and the effect size of the optimal cut-off. As it offers different analysis modes for cut-off point determination depending on the biomarker, the assay and the clinical application, it seemed most feasible to us to use this tool rather than determining the cut-off by receiver operating characteristic curve analysis for example. Our analysis revealed, that patients with a VAT area above the optimal cut-off of 241.4cm^2^ displayed an impaired overall survival. In this context, the prognostic impact of VAT area determination exceeded the prognostic relevance of the patients´ BMI.

Even if an increased VAT area impaired overall survival, it did not influence short-term survival. At this point, we are not able to assess the exact causes of death during long-term study follow up, but hypothesize that included patients with an increased VAT area might display an increased rate of cardiovascular complications and that an enlarged VAT area could also contribute to the development of malignant diseases during follow up as previous studies reported [23, 24]. Our data are in line with recent findings in COVID-19 patients. Here, an increase in visceral adipose tissue area was associated with an augmented likelihood of ICU treatment and mechanical ventilation also reflecting a more severe long-term course of disease [25]. Moreover, our hypothesis is stressed by the fact that diabetes mellitus type 2 patients had more pronounced VAT as well as SAT areas and the VAT area correlated with higher glucose and insulin concentrations. These data are in line with recent data showing a significant association of increased VAT area and insulin resistance [26, 27].

However, in contrast to our findings, previous studies from patients with sepsis, acute respiratory distress syndrome (ARDS), heart failure, and chronic renal failure have shown a decreased mortality in overweight and moderate obesity compared to normal weight critically ill patients [28–31]. The discrepancy between the expected impaired prognosis and the observed rather favorable outcome in these studies at least in patients with mild obesity has been termed “obesity paradox” [32]. Possible explanations for this obesity paradox in critically ill patients are only poorly understood. First, conflicting results from recent meta-analysis and our analysis revealing an association between impaired mortality and obesity need to be interpreted with caution, since a prove of association in such analysis should not be mistaken as prove of causality. Second, different authors have postulated that patients with obesity may have higher reserves and might therefore be more resilient to catabolic states such as critical illness or sepsis [33]. In addition, the adipose tissues and especially the VAT might be involved in the regulation of systemic inflammatory responses as increased volumes of adipose tissue might secrete higher levels of immunomodulatory mediators that could improve survival during critical illness [34]. However, in our cohort, the VAT area negatively correlated with serum levels of cortisol—another mediator that was previously described to convert anti-inflammatory functions that could be beneficial in critically ill patients. Moreover, patients fulfilling sepsis criteria had an increased VAT area even if it was not associated with a hyperinflammatory response measured by leukocyte count, CRP, PCT or IL-6. The fact that the VAT area is not necessarily associated with an augmented systemic response has already been described in previous studies [35]: Here, the authors state that the expression of inflammatory genes is substantially higher in patients with increased SAT compared to VAT area which is in line with our study data. Nevertheless, our study is unable to provide molecular mechanisms triggered by or associated with increased VAT areas in the different subgroups of critically ill patients. It needs to be stressed at this point that investigations on obesity in critically ill patients face a complex pathophysiological situation. We therefore argue that future studies addressing obesity in ICU-patients should consider the underlying disease condition of critical illness as well as metabolic and endocrine characteristics of different adipose tissue compartments resulting in different phenotypes of obesity. Those might subsequently be associated with beneficial or disadvantageous effects on the patients´ outcome.

Another unexpected finding of our study is the strong correlation between the SAT area and levels of transaminases reflecting hepatic inflammation. In contrast, previous studies stated that due to the anatomic link of the VAT and the liver, visceral obesity represents an important pathogenic factor in initiation and progression of fatty liver disease [36]. In our analysis, the SAT area was determined at the height of the 3^rd^ lumbar vertebra, which is referred to as the lower SAT area and does not include analysis of the all portions of SAT. As it is known, different adipose tissue compartments are characterized by differentially activated biological processes such as lipolysis, glucose metabolism, chemokine and hormone release, and gene expression [37]. Since our study does not confirm previous data on the *visceral* adipose tissue to mainly impact fat deposition in the liver and development of steatohepatitis, further studies should address in more detail biological differences of VAT and SAT.

Although remarkable progress in diagnosis and treatment of ICU-patients had been made, the risk stratification as well as diagnostic and therapeutic management during the first days of ICU-admission still embodies a major challenge. The swiftness and accuracy of the initial decisions are essential for the patients’ fortune as the outcome of for instance septic disease or cardiogenic shock depends on early treatment launch. In this context, the use of innovative biomarkers that enable prompt decision-making may especially improve the prognosis of critically ill patients. We agree that a CT scan due to radiation and high costs is not indicated for body composition determination only; however, as a relevant proportion of patients receives a CT scan during ICU admission we claim that the analysis of the body composition including the distribution of adipose tissue in critically ill patients could represent an innovative approach for risk stratification and prognosis prediction. We here have analysed a moderately large but adequately powered cohort of critically ill patients that was well balanced in terms of baseline characteristics, disease etiologies and severity of critical illness. Nevertheless, our study faces some limitations: First, we applied a monocentric design leading to a potential lack of generalizability of results due to selection bias. Second, no longitudinal measurements were performed. Therefore, our study does not meet evidence that adipose tissue areas might be a relevant follow-up diagnostic in critical illness. Moreover, we are unable to provide a molecular mechanism that explains e.g. an increased VAT in sepsis patients. Finally, we were unable to evaluate the exact cause of death with regard to OS and thus a possible causal correlation between increased VAT and decreased OS is missing. However, considering all these limitations, our study supports a potential prognostic value of the VAT area in the context of critically ill patients. Additional investigations need to confirm that such data derived from routine imaging might in the future provide important information for early clinical decision making on patients in emergency departments or intensive care units. To allow a final evaluation of the predictive value of the CT-based assessed VAT area, further studies are needed to address metabolic and immunomodulatory influences in critical illness driven by different adipose tissue compartments.

## Supporting information

S1 File (PDF)

## Abbreviations

ICU: medical intensive care unit
APACHE II: acute physiology and chronic health evaluation score II
SOFA: sequential organ failure assessment score
SAPS II: simplified acute physiology score II
BMI: body mass index
CAD: coronary artery disease
COPD: chronic obstructive pulmonary disease
CRP: C-reactive protein
GFR: glomerular filtration rate
AST: aspartate aminotransferase
ALT: alanine aminotransferase
yGT: gamma glutamyltransferase
BNP: brain natriuretic peptide
LDH: lactate dehydrogenase
HbA1c: glycosylated hemoglobin
OS: overall survival
VAT: visceral adipose tissue
SAT: subcutaneous adipose tissue
