# Supplementary material for: CT-based determination of excessive visceral adipose tissue is associated with an impaired survival in critically ill patients
Source: PLoS One. 2021 Apr 16;16(4):e0250321. doi: 10.1371/journal.pone.0250321 (PMC8051769; doi:10.1371/journal.pone.0250321)
Supplement: S1 File — (PDF) [file pone.0250321.s001.pdf]

CT-based determination of excessive visceral adipose tissue is associated with an impaired survival in critically ill patients

Theresa H. Wirtz[1,*], Sven H. Loosen[2,*], Maximilian Schulze-Hagen[3], Ralf Weiskirchen[4], Lukas Buendgens[1], Samira Abu Jhaisha[1], Philipp Hohlstein[1], Jonathan F. Brozat[1], Tobias Puengel[1,5], Mihael Vucur[2], Pia Paffenholz[6], Christiane Kuhl[3], Frank Tacke[5], Christian Trautwein[1], Tom Luedde[2,#], Christoph Roderburg[5,#], Alexander Koch[1,#]

[1] Department of Medicine III, University Hospital RWTH Aachen, Pauwelsstraße 30, 52074 Aachen, Germany

[2] Clinic for Gastroenterology, Hepatology and Infectious Diseases, University Hospital Düsseldorf, Medical Faculty of Heinrich Heine University Düsseldorf, Moorenstraße 5, 40225 Düsseldorf, Germany

[3] Department of Diagnostic and Interventional Radiology, University Hospital RWTH Aachen, Pauwelsstraße 30, 52074 Aachen, Germany

[4] Institute of Molecular Pathobiochemistry, Experimental Gene Therapy and Clinical Chemistry, University Hospital RWTH Aachen, Pauwelsstraße 30, 52074 Aachen, Germany

[5] Department of Hepatology and Gastroenterology, Charité University Medicine Berlin, Augustenburger Platz 1, 13353 Berlin, Germany

[6] Department of Urology, University Hospital Cologne, Kerpener Straße 62, 50937 Cologne, Germany

[*] These authors share first authorship

[#] These authors share last authorship

Correspondence:

Alexander Koch, M.D.
Department of Medicine III
RWTH-University Hospital Aachen
Pauwelsstraße 30
52074 Aachen
Germany
Phone: +49-241-80-80860
Fax: +49-241-80-82455
Email: akoch@ukaachen.de

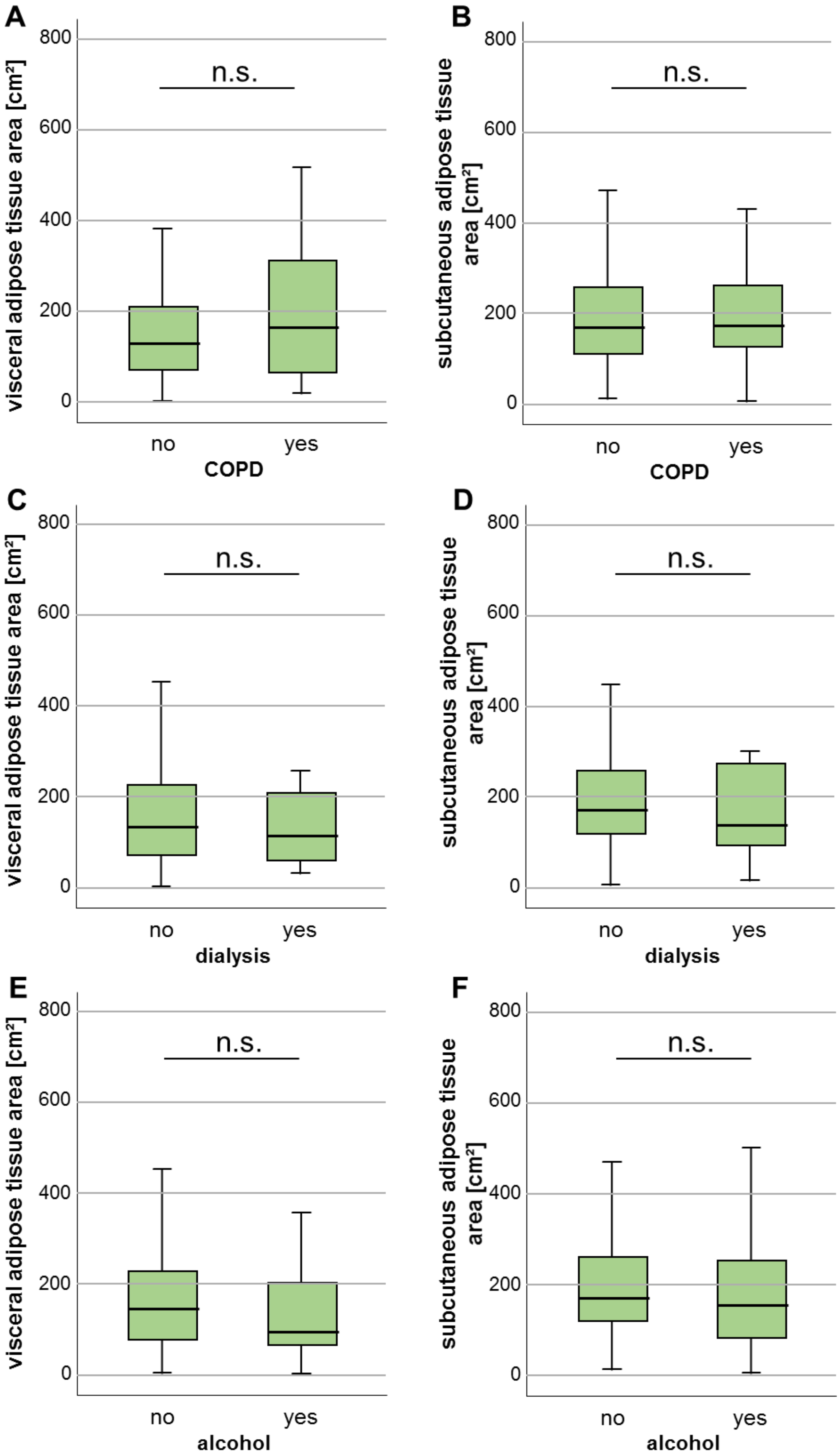

Supplemental Figure 1. Adipose tissue areas and previous medical diseases of critically ill patients. Critically ill patients with previous manifestation of chronic obstructive pulmonary disease (COPD) (A, B), dialysis (C, D) or regular alcohol intake (E, F) do not show differences of both visceral as well as subcutaneous adipose tissue areas.

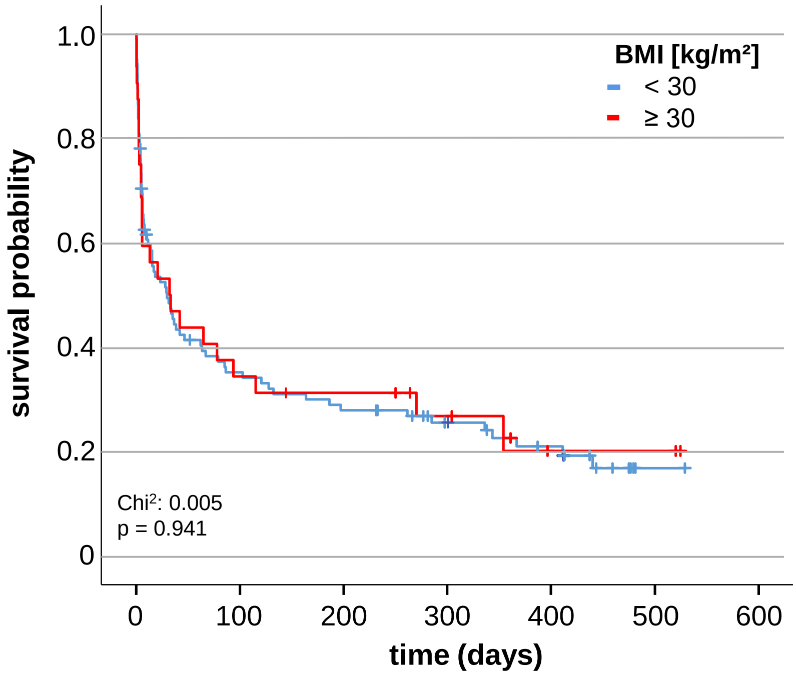

Supplemental Figure 2. Critically ill patients with a BMI below or above 30kg/m² do not show differences in overall survival compared to patients with a BMI below that cut-off. When using a cut-off of 30kg/m² of patients´ BMI values, a BMI >30kg/m² does not predict overall survival as Kaplan Meier curve analysis reveals.
